# 

**Published:** 1839-01-01

**Authors:** 


FISHER, SON & Co. LONDON & PARIS.

THE

### MEDICO-CHIRURGICAL

### REVIEW,

AND

### JOURNAL

OF

### PRACTICAL MEDICINE.

*(NEW SERIES.)*

VOLUME THIRTY,

[1st of OCTOBER 1838 to 31st of MARCH,]

1839.

VOL. X. of DECENNIAL SERIES.

EDITED

BY JAMES JOHNSON, M.D.

PHYSICIAN EXTRAORDINARY TO THE LATE KING,

AND

HENRY JAMES JOHNSON, ESQ.

LECTURER ON ANATOMY AT THE SCHOOL OF ST. GEORGE'S HOSPITAL IN KINNERTON STREET.

Surgeon Genl's Office LIBRARY. Washington, D.C.
57470

LONDON:

PUBLISHED BY S. HIGHLEY, 32, FLEET STREET,

*Re-printed in New York, by Mr. Wood.*

1839.

WI
ME 75 T

PRINTED BY F. HAYDEN,

Little College Street, Westminster.

### BIBLIOGRAPHICAL RECORD.

1. Guy's Hospital Reports. No. VII. Oct. 1838. GEO. H. BARLOW, M.A. and JAMES P. BABINGTON, M.A. &c. 1838.

---

2. The Medical Portrait Gallery. By THOMAS JOSEPH PETTIGREW, Esq. Part 8. Price 3s. Fisher and Son. 1838.

☞ *This Part concludes the life of Dr. Baron, and exhibits portraits and biographies of Dr. Baillie, Dr. Bright, Sir B. Brodie. The first two are good likenesses; but that of Sir Benjamin Brodie, is much more like Mr. Babington than Sir Benjamin. We think it must have been done in a mistake.*

3. The Physiognomy of Mental Diseases. By Sir ALEXANDER MORISON, M.D. No. 6. Monomania, with Love. Oct. 1838. No, 8, Monomania, with Grief. Dec. 1838. Price 3s. 6d. each.

---

4. Transactions of the Medical and Physical Society of Bombay. Vol. 1. Bombay, American Mission Press. 1838. pp. 370. Richardson, Cornhill.

---

5. Urinary diseases and their Treatment. By ROBERT WILLIS, M.D. Physician to the Royal Infirmary for Diseases of Children, &c. 8vo. pp. 408. Sherwood, 1838.

6. Researches on Suppuration. By George Gulliver, Esq. Assistant Surgeon to the Royal Regiment of Horse Guards.

---

7. Report on the Malignant Fever, called the Pali Plague, which prevailed in some parts of Rajapootana, since the month of July, 1836. Prepared and published by order of the Government, &c. By James Ranken, M.D. Calcutta, 1838.

---

8. The Principles of Surgery. Vols. I. and II. containing the Doctrine and Practice relating to Inflammation and its various consequences, Tumours, Wounds, and the states connected with them,—the Surgical Anatomy of the Human Body, and its application to Injuries and Operations. By John Burns, M.D, F.R.S. Regius Professor of Surgery in the University of Glasgow. London, Longman and Co. October, 1838.

---

9. The Surgical Anatomy of the Perinæum. By Thomas Morton, formerly one of the House Surgeons of the University College Hospital. Illustrated with Lithographic Plates and Wood Engravings. 8vo. pp. 80. Taylor and Co. 1838.

---

10. The India Review, and Journal of Foreign Science, &c. Vol. II. No. 22. Edited by F. Corbyn, Esq.

*** *In Exchange.*

---

11. The India Journal of Medical and Physical Science. Edited by F. Corbyn, Esq. New Series. No. II. February, 1838.

*** *In Exchange.*

---

12. On the Successful Treatment of Consumptive Disorders, and Female Complaints connected therewith ;—on Scrofulous Diseases ; and on the Management of Delicate Health by Diet and Regimen, with Cases. By J. J. Furnivall, M.D. late Senior Physician to the Western Dispensary in London. 8vo. pp. 320. London, Nov. 1838.

---

13. Clinical Lecture on the Primary Treatment of Injuries, delivered at the New York Hospital. By Alex. H. Stevens, M.D. 8vo. pp. 34. New York, 1838.

---

14. Lectures on Lithotomy, delivered at the New York Hospital, Dec. 1837. By Alex. H. Stevens, M.D. 8vo. pp. 93. New York, 1838.

---

15. Practical and Surgical Anatomy. By W. J. Erasmus Wilson, Lecturer, &c. Illustrated with fifty engravings on Wood by Bag. 8vo. Longman and Co. 1838.

---

16. A History of British Birds. By W. Yarrell, F.L.S. &c. Illustrated by woodcuts. Part II. Price 2s. 6d. Van Voorst, Nov. 1838.

---

17. Diet and Regimen, Physical, Intellectual, and Moral, as means in the Prevention and Cure of Disease. By Robert Dick, M.D. 8vo. pp. 386. J. Lymington and Co. Glasgow, Nov. 1838.

---

18. Observations on the Oriental Plague and on Quarantines as a means of arresting its progress. Addressed to the British Association assembled at Newcastle on Tyne. By John Bowring. Edinburgh, 1838. 8vo. pp. 45.

☞ *In our next.*

---

19. Practical Observations on the Causes and Treatment of Curvatures of the Spine, with Hygienic Directions, &c. By Samuel Hare, Surgeon. 8vo. pp. 151. Plates. Simpkin and Co. London, Nov. 1838.

---

20. Medical Portrait Gallery. Part 9, price 3s. By Mr. Pettigrew, containing Memoirs of Sir B. Brodie, John Hunter, and Mr. W. Lawrence. Nov. 1838.

---

21. Outlines of Military Surgery. By Sir George Ballingall, M.D. Second Edition, Edinb. 1838.

*** *In our next.*

---

22. Treatises on Physiology and Phrenology :—from the Seventh Edition of the Encyclopædia Britannica. By P. M. Roget, M.D. &c. In Two Volumes. 8vo. Edinburgh, 1838.

---

23. A Statistical Report of Addenbroke's Hospital, for the year 1837. From the Transactions of the Cambridge Philosophical Society, Vol. VI. Part III. By Henry J. Bond, M.D. 4to. 1838.

---

24. The Chirurgico-Comico Alphabet, complete in 24 plates. By Pill-Box. Renshaw, Strand. 1838. Price 4s. 6d. *Very droll.*

---

25. A Treatise on the Structure, Economy and Diseases of the Ear; being the Essay for which the Fothergillian Medal was awarded by the Medical Society of London. By Geo. Pilcher, Lecturer on Anatomy and Surgery, &c. &c. 8vo, pp. 324, with plates. Highley, Nov. 1838.

26. On the Causes of Epidemic Fever in the Metropolis, more especially as regards the Condition of the Labouring Classes. By W. Holt Yates, M.D. Physician to the General Dispensary. 8vo, pp. 30. Edwards, Ave-Maria Lane. Dec. 1838.

27. On the Objects and mutual Relations of the Medical Sciences; an Introductory Address delivered at the Middlesex Hospital School of Medicine, Oct. 1838. By Fred. S. Leighton, M.D. Lecturer on Forensic Medicine. Renshaw, Nov. 1838.

28 An Introductory Address on the Studies for the Medical Profession. Addressed to the Medical School of St. George's Hospital, October, 1838. By Sir B. C. Brodie, Bart. F.R.S. &c. London, 1838.

29. A Letter to the Right Honourable the Lord Chancellor on the Present State of the Law of Lunacy, with Suggestions, &c. By a Barrister of the Inner Temple. W. Crofts. pp. 16. Nov. 1838.

*** *In our next.*

30. The Medical Annual; or British Medical Almanac, 1839. Edited by William Farr. Sherwood and Co. 1838. Price three shillings.

31. Lectures on the Physiology and Diseases of the Chest, &c. By Charles J. B. Williams, M.D. F.R.S. Illustrated by Engravings. pp. 204. 1838. No publisher.

32. A Text-Book of Human Anatomy, designed to facilitate the Study of that Science. By Robert Hunter, M.D. Professor of Anatomy, Andersonian College, Glasgow. Second Edition, Glasgow, 1838.

33. The Medical Portrait Gallery, Part 10. Price 3s. By T. J. Pettigrew, Esq. *Contents.*—Conclusion of W. Lawrence's Biography—Memoir and Portrait of Sir John Pringle—ditto, Dr. Clutterbuck.

*** *The letter-press is ably executed in this Part, but we think the portrait of Dr. Clutterbuck, though a good engraving, is not a very striking likeness.*

34. Physiological Explanation of the Beauty of Form. By Benjamin Joslyn, M.D. Professor of Natural Philosophy, &c. in Union College, N.Y. Albany, 1837. pp. 30, with a plate.

35. The Medical Pocket Book and Almanac for 1839. By John Foote. Renshaw, 1838.

*** *This little book contains a number of good things; but as just half of it is blank ruled paper, just half its utility is sacrificed. We would strongly advise Mr. Foote to remedy this defect next year.*

36. An Account of the Proceedings at the Sixth Anniversary Meeting of the Provincial Medical and Surgical Association, held at Bath, Wednesday and Thursday, July 18 and 19, 1838. pp. 100, Worcester, 1838.

37. A Lecture, introductory to the Business of the Original School of Medicine, Peter-street; delivered by G. T. Hayden, A.B. M.R.C.S.I. Surgeon to the Anglesey Lying-in Hospital, and Ophthalmic Infirmary, and Lecturer on Anatomy and Surgery. Dublin, Fannin and Co.; London, Renshaw, 1838.

38. Principles of General and Comparative Physiology, intended as an Introduction to the Study of Human Physiology, and as a Guide to the Philosophical Pursuit of Natural History. By Wm. B. Carpenter, M.R.C.S. &c. Lecturer on Forensic Medicine in Bristol. 8vo, pp. 478—six plates. Churchill, London, Dec. 1838.

39. Manual of Descriptive and Pathological Anatomy, by J. F. Meckel, Professor of Anatomy at Halle, &c. Translated from the German into French, with Additions and Notes, by A. J. L. Jourdan, Mem. of Royal Acad. of Medicine at Paris, &c. and G. Breschet, Adjunct Prof. of Anatomy at the School of Medicine, &c. Translated from the French, with Notes, by A. S. Doane, A.M. M.D. and others. In 2 vols. 12mo. pp. 571-650. London, Henderson, 1838.

40. The Student's Guide to the Hospitals and Medical Institutions of Paris. To which is added an Outline of the Edinburgh and German Universities. By John Wiblin, M.R.C.S. Small octavo, pp. 70. Renshaw, 1838.

*** *A most admirable and useful guide.*

41. The Philosophy of Disease; or an Outline of the Principles of Medical Science, comprising a Brief Exposition of the Laws of Inflammatory Action. By James Bower Harrison, M.R.C.S. in London. Small 8vo. pp. 152. Simpkin and Marshall, Dec. 1838.

